# A call to integrate mental health support into Sickle Cell Disease care: The value of task-sharing in low- and middle-income countries

**DOI:** 10.1371/journal.pmen.0000360

**Published:** 2025-07-03

**Authors:** John Patena, Alden Yuanhong Lai, Annika C. Sweetland, Joyce Gyamfi, Emmanuel Peprah

**Affiliations:** 1 Implementing Sustainable Evidence-based interventions through Engagement (ISEE) Lab, Department of Global and Environmental Health, New York University School of Global Public Health, New York City, New York, United States of America; 2 Department of Public Health Policy and Management, New York University School of Global Public Health, New York City, New York, United States of America; 3 Department of Psychiatry, Columbia University Vagelos College of Physicians and Surgeons/New York State Psychiatric Institute, New York City, New York, United States of America; PLOS: Public Library of Science, UNITED KINGDOM OF GREAT BRITAIN AND NORTHERN IRELAND

## Existing mental health care for sickle cell disease is sparse

Sickle Cell Disease (SCD) disproportionately affects people in low- and middle-income countries (LMIC) [1]. People living with SCD experience high rates of depressive and anxiety symptoms, a topic that is rarely addressed in SCD care [2]. Current guidelines for SCD management lack a comprehensive approach to address the mental health needs of people living with this condition [3,4]. The omission of mental health considerations is a significant oversight. Mental health conditions profoundly affect quality of life, negatively impact healthcare seeking behavior, and can exacerbate disease outcomes if left unaddressed. Closing this gap requires integrating mental health services into existing SCD care models, ensuring a truly comprehensive approach that meets the physical, psychological, and emotional needs of people living with SCD.

## Task-sharing mental health interventions as a solution

Task-sharing – a well-utilized strategy that redistributes care traditionally provided by mental health specialists to non-mental health specialists – has proven effective in expanding access to mental health services in low-resource settings [5]. Problem-Solving Therapy (PST) offers a promising avenue for addressing depression and anxiety in SCD care. Research demonstrates PST’s effectiveness in managing depression and anxiety for patients with other chronic conditions in LMICs with notable success [6]. One task-sharing intervention which incorporates principles of PST is the *Friendship Bench*. Originally developed for use in Zimbabwe and implemented in other LMICs, the Friendship Bench has been shown to effectively reduce depression and anxiety, leveraging trusted lay health workers (LHW) to provide culturally adaptable, community-supported mental health interventions [7]. Its success is driven by strong community and political buy-in, as well as its feasibility and acceptability broadly in LMICs. Given these strengths, the Friendship Bench provides a valuable foundation for developing novel task-sharing mental health interventions tailored to the needs of people living with SCD.

## Considerations to implement task-sharing mental health interventions for SCD

Task-sharing mental health interventions represent a low-cost and effective alternative for addressing the mental health needs of people living with SCD, particularly in low-resource settings. The Friendship Bench is designed to provide low-intensity psychological support for those experiencing mild to moderate symptoms of depression, which will likely meet the needs of most people living with SCD who experience lower severity levels of depression [8]. However, these interventions are only part of a larger, multi-tiered solution. Task-sharing mental health interventions can function as an entry point into the broader mental health care system. Communities that have limited resources for formal mental health services (e.g., psychiatrists, psychologists), can utilize task-sharing as a way to provide psychoeducation and social support. Individuals who may need more comprehensive mental health services (e.g., long-term therapy, pharmacological treatment) will still receive basic mental health support until more care can be provided.

The strongest predictor of success for task-sharing mental health interventions are the personal characteristics of the task-sharing providers (e.g., LHW) [9]. Research has consistently shown that the effectiveness of such interventions are closely tied to the LHWs’ ability to build trust and rapport with the communities they serve. Being perceived as family-like figures, sharing similar social and economic challenges as patients, and understanding local cultural norms and values significantly enhance their credibility and effectiveness.

## Alignment with global health efforts

Improving mental health care into SCD management aligns with global health initiatives promoting the integration of mental health services into primary care. The World Health Organization has long advocated for a collaborative care model that integrates mental health treatment into the broader healthcare system [10]. This approach ensures that mental health care is not isolated but seamlessly integrated into the continuum of care for chronic diseases. In September 2025, the United Nations will host the *Fourth High-Level Meeting on Noncommunicable Diseases and Mental Health* to discuss, define, and commit to actionable recommendations for integrating noncommunicable diseases (NCD) and mental health care into national health financing and policy frameworks. This provides an important opportunity to advocate for the inclusion of SCD in global health priorities [10]. By leveraging these global discussions and frameworks, advocates for SCD care can build momentum for more comprehensive, integrated approaches to addressing both physical and mental health challenges in SCD populations.

## Conclusion

We strongly advocate for the urgent adoption and integration of such interventions to support the mental health of people living with SCD. Beyond improving individual well-being, this approach promises to enhance overall health outcomes, reduce healthcare costs, and contribute to more holistic and equitable care for people living with SCD. It is time for global health stakeholders to prioritize mental health as a core component of SCD care, ensuring no patient is left behind and has optimal quality of life.
